# Regulatory T cell frequency, but not plasma IL-33 levels, represents potential immunological biomarker to predict clinical response to intravenous immunoglobulin therapy

**DOI:** 10.1186/s12974-017-0818-5

**Published:** 2017-03-20

**Authors:** Mohan S. Maddur, Emmanuel Stephen-Victor, Mrinmoy Das, Praveen Prakhar, Varun K. Sharma, Vikas Singh, Magalie Rabin, Jamma Trinath, Kithiganahalli N. Balaji, Francis Bolgert, Jean-Michel Vallat, Laurent Magy, Srini V. Kaveri, Jagadeesh Bayry

**Affiliations:** 1 Institut National de la Santé et de la Recherche Médicale, Unité 1138, Paris, 75006 France; 2 Centre de Recherche des Cordeliers, Equipe— Immunopathologie et immuno-intervention thérapeutique, Paris, 75006 France; 3 Sorbonne Universités, UPMC Univ Paris 06, UMR S 1138, Paris, 75006 France; 4 Université Paris Descartes, UMR S 1138, Paris, 75006 France; 5 Emory Vaccine Center, Yerkes National Primate Research Center, Emory University, Atlanta, GA 30329 USA; 6 Department of Microbiology and Cell Biology, Indian Institute of Science, Bangalore, 560012 India; 7 Réanimation Neurologique, Neurologie 1, Hôpital de la Pitié-Salpêtrière, Paris, 75651 France; 8 Centre de Référence ‘Neuropathies Périphériques Rares’ et Service de Neurologie, Hôpital Universitaire Limoges, Limoges, 87042 France

**Keywords:** IVIG, Guillain-Barré syndrome, T_reg_ cells, IL-33, TNF-α, Clinical response, Regulatory T cells

## Abstract

**Background:**

Intravenous immunoglobulin (IVIG) is a polyspecific pooled immunoglobulin G preparation and one of the commonly used therapeutics for autoimmune diseases including those of neurological origin. A recent report in murine model proposed that IVIG expands regulatory T (T_reg_) cells via induction of interleukin 33 (IL-33). However, translational insight on these observations is lacking.

**Methods:**

Ten newly diagnosed Guillain-Barré syndrome (GBS) patients were treated with IVIG at the rate of 0.4 g/kg for three to five consecutive days. Clinical evaluation for muscular weakness was performed by Medical Research Council (MRC) and modified Rankin scoring (MRS) system. Heparinized blood samples were collected before and 1, 2, and 4–5 weeks post-IVIG therapy. Peripheral blood mononuclear cells were stained for surface CD4 and intracellular Foxp3, IFN-γ, and tumor necrosis factor alpha (TNF-α) and were analyzed by flow cytometry. IL-33 and prostaglandin E2 in the plasma were measured by ELISA.

**Results:**

The fold changes in plasma IL-33 at week 1 showed no correlation with the MRC and MRS scores at weeks 1, 2, and ≥4 post-IVIG therapy. Clinical recovery following IVIG therapy appears to be associated with T_reg_ cell response. Contrary to murine study, there was no association between the fold changes in IL-33 at week 1 and T_reg_ cell frequency at weeks 1, 2, and ≥4 post-IVIG therapy. T_reg_ cell-mediated clinical response to IVIG therapy in GBS patients was associated with reciprocal regulation of effector T cells-expressing TNF-α.

**Conclusion:**

T_reg_ cell expansion by IVIG in patients with autoimmune diseases lack correlation with IL-33. T_reg_ cell frequency, but not plasma IL-33 levels, represents potential immunological biomarker to predict clinical response to IVIG therapy.

## Findings

### Introduction

Intravenous immunoglobulin (IVIG) is a polyspecific pooled immunoglobulin G preparation and one of the commonly used immunotherapeutics for the treatment of autoimmune diseases including those of neurological origin [1–4]. Various reports demonstrate that IVIG exerts beneficial effects through several mutually non-exclusive mechanisms including expansion of CD4^+^Foxp3^+^ regulatory T cells (T_reg_ cells) [5–10]. Some of these mechanisms are also being explored as biomarkers of IVIG response [11, 12]. T_reg_ cells play an indispensable role in the maintenance of immune tolerance and suppress unnecessary deleterious immune responses, such as autoimmunity [13]. IVIG is shown to expand T_reg_ cells and enhance their functions in experimental animal models and patients with autoimmune diseases [9, 14–17]. Recent studies suggested that IVIG-induced expansion of T_reg_ cells requires the prime role of dendritic cells (DCs), and involves interaction of IVIG with C-type lectin receptors, such as dendritic cell-specific intercellular adhesion molecule-3-grabbing non-integrin (DC-SIGN) and dendritic cell immunoreceptor (DCIR), and is mediated through prostaglandin (PG) E_2_ secreted by DCs [15, 18]. Interestingly, based on the murine models of autoimmune diseases, i.e., antibody-mediated K/BxN arthritis and T cell-mediated experimental autoimmune encephalitis, a recent report proposed that induction of interleukin 33 (IL-33) in macrophages by IVIG through interaction of α2,6-sialylated crystallizable fraction (Fc) with SIGN-R1 or human DC-SIGN is essential for the expansion of T_reg_ cells [19, 20]. However, translational insight on these observations in patients with autoimmune diseases is lacking.

Guillain-Barré syndrome (GBS) is an autoimmune disease of neurological origin affecting the peripheral nerves [21]. GBS is believed to be caused by effector T cells and autoantibodies to myelin components [22, 23]. Furthermore, GBS patients display reduced frequency of T_reg_ cells that are required for the prevention of autoimmunity [24]. Amelioration of experimental autoimmune neuritis, an experimental model of GBS, was associated with up-regulation of T_reg_ cells [25]. Currently IVIG is used as a first-line therapy for GBS [26]. Of note, sialylated IVIG has been shown to suppress anti-ganglioside antibody-mediated nerve injury in experimental GBS model and was associated with increased expression of IL-33 mRNA [27]. Sialylated IVIG also inhibited anti-ganglioside antibody-mediated complement deposition in vitro [28]. In view of these reports, we investigated the role of IL-33 towards clinical response to IVIG treatment and T_reg_ cell expansion in GBS patients. We found that kinetics of peripheral T_reg_ cell expansion and improvement of clinical symptoms in GBS patients following IVIG therapy lack correlation with the level of induction of IL-33 in the blood.

## Materials and methods

### Clinical evaluation and sample collection

A total of ten treatment-naïve patients (mean age of 68 years) exhibiting neurological signs of GBS were enrolled based on the diagnostic criteria (Table 1). The study was approved by relevant ethical committee (84-2012-08, CHU Limoges) and consent was obtained from the enrolled patients. The patients received IVIG at the rate of 0.4 g/kg for three (three patients) or five (seven patients) consecutive days. Clinical evaluation for the muscular weakness using Medical Research Council (MRC) and modified Rankin score (MRS) grading systems and collection of heparinized blood samples were done before and 1, 2, and 4–5 weeks after the initiation of IVIG treatment (post-IVIG) [29]. For all the patients, the MRC is a sum score of ten muscle groups tested bilaterally (min = 0, max = 100).

**Table 1:** Demographics and clinical information of GBS patients

Sl no.	Age (years)	Sex	MRC score (pre-IVIG)	MRS (pre-IVIG)
P1	61	Male	30	5
P2	68	Female	80	4
P3	57	Male	85	1
P4	70	Female	68	4
P5	82	Male	70	3
P6	66	Female	78	2
P7	85	Male	54	4
P8	60	Female	70	4
P9	68	Male	54	5
P10	59	Female	13	4

### Flow cytometry and ELISA

Plasma and peripheral blood mononuclear cells (PBMCs) were separated from the heparinized blood samples. PBMCs were stained for surface CD4 and intracellular Foxp3, and also interferon gamma (IFN-γ) and tumor necrosis factor alpha (TNF-α) following stimulation with phorbol myristate acetate (50 ng/mL) and ionomycin (500 ng/mL, Sigma-Aldrich, France), along with GolgiStop (BD Biosciences, France), for 4 h. The intracellular staining was performed using Foxp3 staining kit (eBioscience, France) as per the manufacturer’s instructions. Stained cells were acquired on LSR II (BD Biosciences) flow cytometer and data was analyzed using BD FACS DIVA (BD Biosciences) and FlowJo (FlowJo LLC, USA) softwares. IL-33 was measured in the plasma by ELISA (R&D systems, France). The amount of PGE_2_ in the plasma samples was estimated by ELISA as described previously [15].

### Statistical analysis

One-way ANOVA “Kruskal-Wallis test” was used for the analysis of IL-33 in the plasma. Pearson correlation (*r*) was used to determine the association between MRC or MRS scores and plasma levels of IL-33 and PGE_2_ and the frequency of T_reg_ cells in the blood of GBS patients at different time points following IVIG treatment. Same assay was also used for determining the association between frequency of T_reg_ cells and CD4^+^IFN-γ^+^ and CD4^+^TNF-α^+^ T cells.

## Results and discussion

**Fig. 1:**
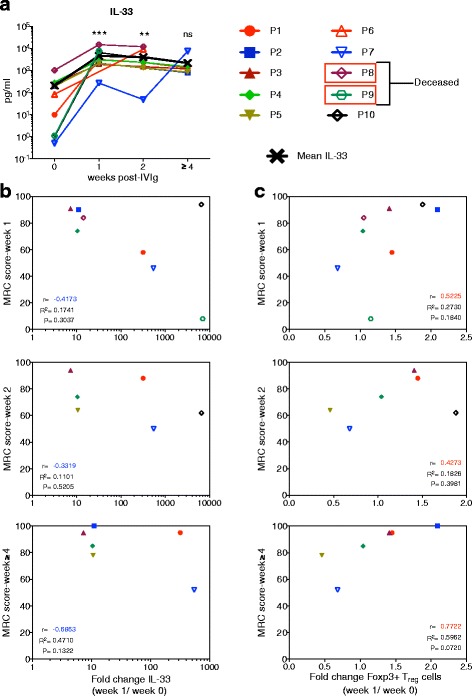
Correlation between changes in the plasma IL-33 and regulatory T (T_reg_) cell frequencies and clinical response to IVIG therapy in GBS patients. **a** Temporal changes in the amount of IL-33 (pg/ml) in the plasma of ten Guillain-Barré syndrome patients before (week 0) and weeks 1, 2, and ≥4 following initiation of IVIG therapy. Statistical significance as determined by one-way ANOVA. ***P < 0.001, **P < 0.01, ns = not significant. **b** Correlation between the clinical response (MRC score) at different time points (weeks 1, 2, and ≥4) after initiation of IVIG therapy and the fold changes in plasma IL-33 at week 1. **c** Correlation between the clinical response (MRC score) at different time points (weeks 1, 2, and ≥4) after initiation of IVIG therapy and the fold changes in circulating T_reg_ cells at week 1. Each *symbol* represents individual patient. *r* = Pearson correlation. *p* = statistical significance

The overall rate of new incidence of GBS is 0.6–4.0/year/100,000 people. We limited our investigation only to newly diagnosed and treatment-naïve GBS patients to avoid possible modulatory effects of past IVIG therapy or other therapeutic agents on the T_reg_ cells and other immune parameters. This explicates the difficulties in enrolling treatment-naïve patients for the investigation. As kinetics of IL-33 induction in the plasma of patients with autoimmune diseases following IVIG therapy was not explored earlier, we first analyzed plasma levels of IL-33 in ten GBS patients at various time points (pre-IVIG and weeks 1, 2, and ≥4 after initiation of IVIG therapy). Patients had variable pre-IVIG plasma IL-33 levels that significantly increased at week 1 in all the patients. This data thus confirms our recent observation in IVIG-treated inflammatory myopathy and anti-neutrophil cytoplasmic antibody-associated vasculitis patients [30]. At subsequent time points, IL-33 levels in the plasma of IVIG-treated GBS patients declined progressively but remain significantly higher than the pre-IVIG levels (Fig. 1a) indicating sustained effect of IVIG beyond its half-life on plasma IL-33 levels.

In order to explore the role of IL-33 in IVIG-mediated therapeutic effects, we analyzed whether IL-33 induction correlates with clinical response to IVIG therapy and T_reg_ cell expansion [29]. Of note, the fold changes in plasma IL-33 level at week 1 showed no correlation with the MRC scores at weeks 1, 2, and ≥4 post-IVIG therapy (Fig. 1b). Also, the *r* values are consistently negative at all the time points. On the other hand, T_reg_ cell response following IVIG therapy appears to be linked with clinical recovery from GBS and is consistent with the previous observations on essential role of T_reg_ cells in IVIG-induced protection in mouse models [18, 31]. Yet, we observed no correlation between the fold-changes in T_reg_ cells at week 1 and clinical score at weeks 1, 2, and ≥4 (Fig. 1c). These results suggest that T_reg_ cells, but not IL-33, might predict clinical response to IVIG therapy.

Similar observations are also made with MRS parameters. We found that the fold changes in IL-33 level at week 1 displayed no correlation with MRS scores at weeks 1, 2, and ≥4 following initiation of IVIG therapy indicating that clinical improvement as analyzed by MRS is also not associated with changes in IL-33 levels in the blood. As in the case of MRC scores, changes in T_reg_ cells were suggestive of clinical response to IVIG therapy by MRS parameters as well. However, the fold changes in T_reg_ cells at week 1 and MRS scores at weeks 1, 2, and ≥4 post-IVIG therapy did not correlate (Table 2).

**Table 2:** Correlation between MRS scores at different time points (weeks 1, 2, and ≥4) after initiation of IVIG therapy and the immunological parameters such as IL-33, T_reg_ cells, and PGE_2_ at week 1. The values in the parenthesis denote statistical significance

*r* values (*p* values)		MRS (week post-IVIG)		
	Week	1	2	≥4
IL-33	1	0.57 (0.14)	−0.60 (0.20)	0.71 (0.42)
T_reg_ cell	1	−0.37 (0.3601)	−0.61 (0.1067)	−0.47 (0.0684)
PGE_2_	1	−0.16 (0.6954)	−0.29 (0.4361)	−0.07 (0.8936)

**Fig. 2:**
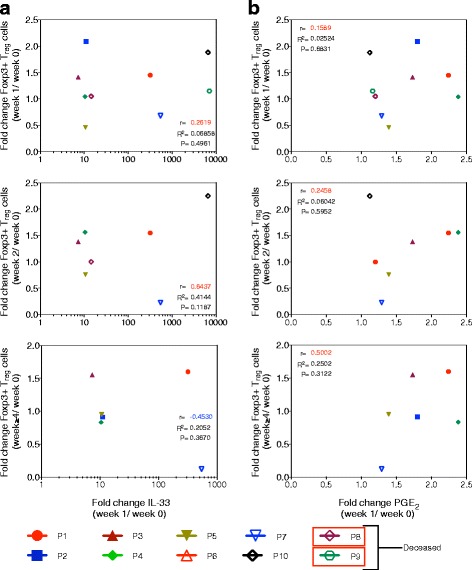
Correlation between changes in plasma IL-33 and PGE_2_ and IVIG-mediated circulating T_reg_ cell expansion in GBS patients. **a** Correlation between the fold changes in circulating T_reg_ cells at different time points (weeks 1, 2, and ≥4) after initiation of IVIG therapy and the fold changes in plasma IL-33 level at week 1. **b** Correlation between the fold changes in circulating T_reg_ cells at different time points (weeks 1, 2, and ≥4) after initiation of IVIG therapy and the fold changes in plasma PGE_2_ level at week 1. Each *symbol* represents individual patient. *r* = Pearson correlation. *p* = statistical significance

As data from the recent mouse study suggested that T_reg_ cell expansion by IVIG is dependent on IL-33 [20], we analyzed correlation between IL-33 and T_reg_ cells in these patients at various time points. Contrary to Fiebiger et al., we found no consistent association between the fold changes in IL-33 level at week 1 and T_reg_ cell frequency at weeks 1, 2, and ≥4 after initiation of IVIG therapy (Fig. 2a), implying that T_reg_ cell expansion by IVIG in patients with autoimmune diseases is not related to levels of IL-33 in the plasma.

**Fig. 3:**
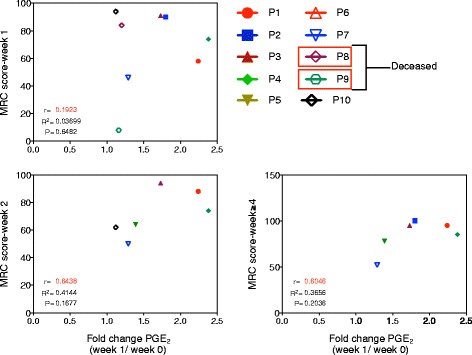
Clinical response to IVIG therapy and its correlation with plasma PGE_2_. Correlation between the clinical response (MRC score) at different time points (weeks 1, 2, and ≥4) after initiation of IVIG therapy and the fold changes in plasma PGE_2_ level at week 1. Each *symbol* represents individual patient. *r* = Pearson correlation. *p* = statistical significance

Expansion of T_reg_ cells in the periphery is mediated by the signals derived from professional antigen presenting cells such as DCs. Unlike other cytokines, IL-33 can be released into the microenvironment only upon injury to the cells and acts as alarmin to signal tissue damage to the immune system [32]. Our recent report shows that DC-SIGN-positive human innate cells derived either from the peripheral blood or from the spleen do not release IL-33 upon IVIG exposure [30]. Therefore, unlike murine models [19, 20], it is likely that damaged non-immune cells like endothelial cells or epithelial cells might have contributed to increased levels of IL-33 observed in the plasma of patients following IVIG therapy. Despite the lack of IL-33 induction, “IVIG-educated” DC-SIGN-positive human DCs induced T_reg_ cell expansion, a process mediated via cyclooxygenase-2-dependent PGE_2_ and independent of Fc-fragments of IVIG [15]. Notably, there was a significant increase in the plasma levels of PGE_2_ in IVIG-treated GBS patients [33]. In the current study, although plasma PGE_2_ levels are enhanced in IVIG-treated patients, we found no correlation between the fold changes in PGE_2_ level at week 1 and T_reg_ cell frequencies at week 1, 2, and ≥4 post-IVIG therapy (Fig. 2b). Similarly, fold changes in PGE_2_ level at week 1 did not correlate with MRC score at weeks 1, 2, and ≥4 post-IVIG therapy (Fig. 3). The reason for the non-significant correlation of values might rests in the low number of patients. A recent report shows that plasma PGE_2_ levels correlate with the prevention of IVIG resistance in Kawasaki disease [34]. Taken together, these results are suggestive of a role for PGE_2_-mediated expansion of T_reg_ cells in the clinical recovery of patients following IVIG therapy.

In the previous report [30], we analyzed IL-33 in the plasma of patients with rheumatic disorders as early as 48–72 h following initiation of IVIG therapy. With the exception of two, the remaining 14 patients had IL-33 levels below 500 pg/ml of plasma following IVIG therapy. Although the underlying pathologies in that report and current study are different, we found that at 1 week following initiation of IVIG therapy, IL-33 level in the plasma was more than 1000 pg/ml in nine GBS patients. Furthermore, in majority of the patients (seven), IVIG was given daily for five consecutive days. Therefore, lack of consistent association between the fold changes in IL-33 level and T_reg_ cell frequency following IVIG therapy might not be due to loss of IL-33 subsequent to proteolytic cleavage and binding to ST2 receptor. Also, IL-33 levels observed at first week are maintained in majority of the patients even after 4 weeks post-IVIG. Whether polymorphisms in either ST2 receptor, IL-1RL1, and/or IL-33 itself, skew the plasma kinetics and/or downstream effects of IL-33 remains to be determined [35, 36].

Current literature suggests that IVIG can induce T_reg_ cell expansion by several mutually non-exclusive mechanisms [9, 14, 16, 17, 31] that may vary among the pathologies and even among the patients with same underlying pathology. This might explain the lack of significant correlation between PGE_2_ and T_reg_ cells. As we restricted our study only to treatment-naïve GBS patients, difficulty to get large number of patients provides an alternative explanation for the lack of significant positive correlation between changes in the IL-33 and PGE_2_, and T_reg_ cells. Role of IL-33 in the microenvironment towards DC-mediated human Treg expansion requires further exploration.

**Fig. 4:**
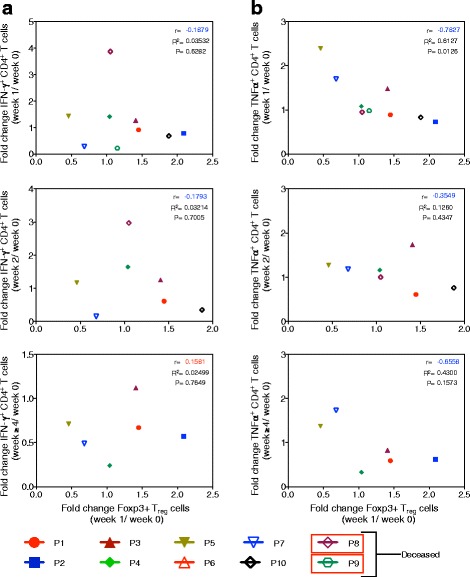
T_reg_ cell response following IVIG therapy in GBS patients correlates negatively with effector T cells. **a** Correlation between the fold changes in circulating T_reg_ cells at week 1 after initiation of IVIG therapy and the fold changes in IFN-γ^+^ CD4^+^ T cells at weeks 1, 2, and ≥4. **b** Correlation between the fold changes in circulating T_reg_ cells at week 1 following initiation of IVIG therapy and the fold changes in TNF-α^+^ CD4^+^ T cells at weeks 1, 2, and ≥4. Each *symbol* represents individual patient. *r* = Pearson correlation. *p* = statistical significance

In addition to expansion of T_reg_ cells, IVIG is also known to enhance their functions and suppress effector T cell proliferation and production of cytokines such as IFN-γ and TNF-α [16, 31]. Interestingly, in IVIG-treated GBS patients, there was a significant negative correlation between the fold changes in T_reg_ cells at week 1 and T cells expressing TNF-α at week 1 post-IVIG therapy. However, such consistent association was not observed with T cells expressing IFN-γ (Fig. 4a, b). Thus, T_reg_ cell-mediated clinical response to IVIG therapy is associated with reciprocal regulation of effector T cells in GBS patients.

## Conclusions

Because of its role as promoter of type 2 immune responses and regulator of innate and adaptive immune responses, IL-33 has been explored as therapeutic option in pre-clinical models of Alzheimer’s disease [37], stroke [38], cerebral malaria [39], and transplantation [40]. In addition, IL-33 was also reported to be essential to attenuate viral-induced encephalitis [41]. In line with these reports, enhanced expression of IL-33 in mice was suggested to mediate T_reg_ cell expansion and protection by IVIG [19, 20, 27]. Translation of these results to human is complicated and requires thorough investigation. Indeed, we found that up-regulation of plasma IL-33 does not correlate with the clinical response to IVIG therapy and expansion of T_reg_ cells in GBS patients. T_reg_ cells on the other hand are negatively correlated to effector T cells expressing TNF-α. Thus, the mechanism of IL-33 induction by IVIG and its role in T_reg_ cell expansion observed in mouse models might not be applicable to human. Small size of the patients’ cohort represents limitation of this study. Nevertheless, based on these findings, T_reg_ cell frequency, but not plasma IL-33 levels, represents potential immunological biomarker to predict clinical response to IVIG therapy.

## Abbreviations

DCIR: Dendritic cell immunoreceptor
DCs: Dendritic cells
DC-SIGN: Dendritic cell-specific intercellular adhesion molecule-3-grabbing non-integrin
GBS: Guillain-Barré syndrome
IFN-γ: Interferon gamma
IL-33: Interleukin 33
IVIG: Intravenous immunoglobulin
MRC score: Medical research council score
MRS: Modified Rankin score
PGE_2_: Prostaglandin E2
TNF-α: Tumor necrosis factor alpha
T_reg_ cells: Foxp3^+^ regulatory T cells
